# Supplementary figures and images for: Colonization of the Caenorhabditis elegans gut with human enteric bacterial pathogens leads to proteostasis disruption that is rescued by butyrate
Source: PLoS Pathog. 2021 May 6;17(5):e1009510. doi: 10.1371/journal.ppat.1009510 (PMC8101752; doi:10.1371/journal.ppat.1009510)

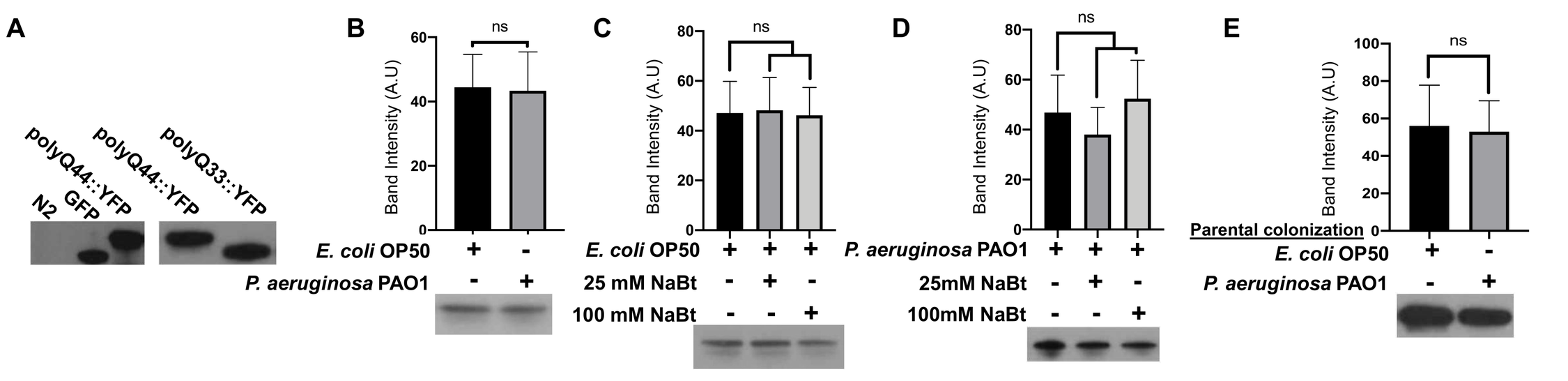

Supplement: S1 Fig — A) Western blot confirmation of antibody specificity. N2: no band; gcs-1p::GFP, polyQ33::YFP, polyQ44::YFP all show bands corresponding to their increasing molecular weight, respectively. B-E) Western blotting and image-J quantification of the soluble fraction of polyQ44::YFP in four-day-old C. elegans colonized with: B) E. coli OP50 and P. aeruginosa PAO1, C) E. coli OP50 with 0, 25, and 100 mM butyrate, D) P. aeruginosa PAO1 with 0, 25, 100 mM butyrate, E) F1 progeny from parental generations colonized with E. coli OP50 and P. aeruginosa PAO1. Band intensity is measured in arbitrary units (A.U). Data are representative of five independent experiments, B; four independent experiments, C and D; three independent experiments, E. Error bars represent SEM. Significance for C and D was calculated using one-way ANOVA followed by multiple comparison Dunnett’s post-hoc test. Significance for B and E was calculated using Student’s t-test (ns = not significant). (TIF) [file ppat.1009510.s001.tif]

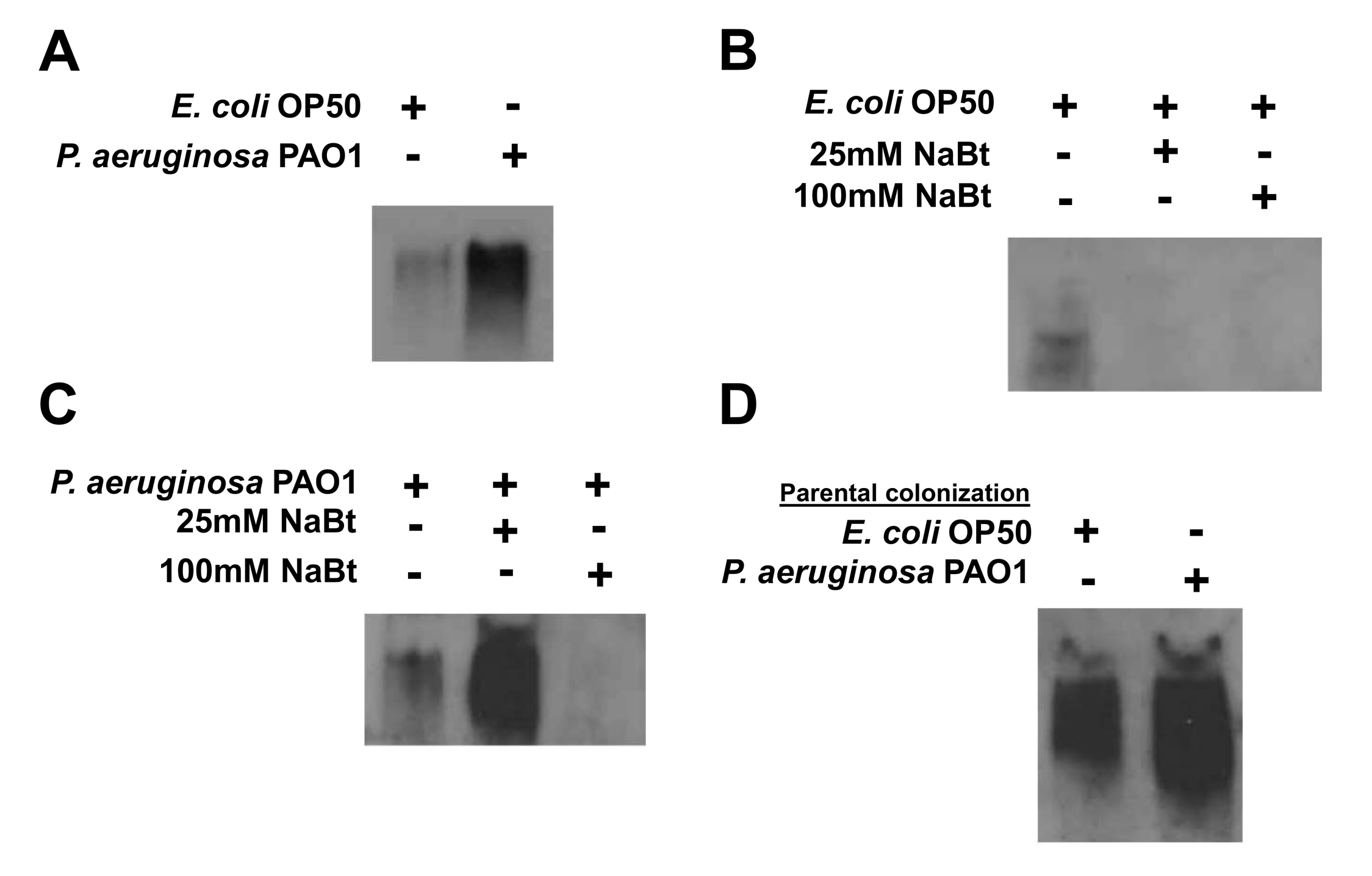

Supplement: S2 Fig — A-C) Insoluble fraction of polyQ44::YFP aggregates in four-day-old C. elegans expressing intestinal polyQ44::YFP colonized with: A) E. coli OP50 and P. aeruginosa PAO1, B) E. coli OP50 in the presence of 0, 25, 100 mM butyrate, and C) P. aeruginosa PAO1 in the presence of 0, 25, 100 mM butyrate. D) insoluble polyQ44 extracted from F1 progeny whose parents were colonized with E. coli OP50 or P. aeruginosa PAO1. (TIF) [file ppat.1009510.s002.tif]

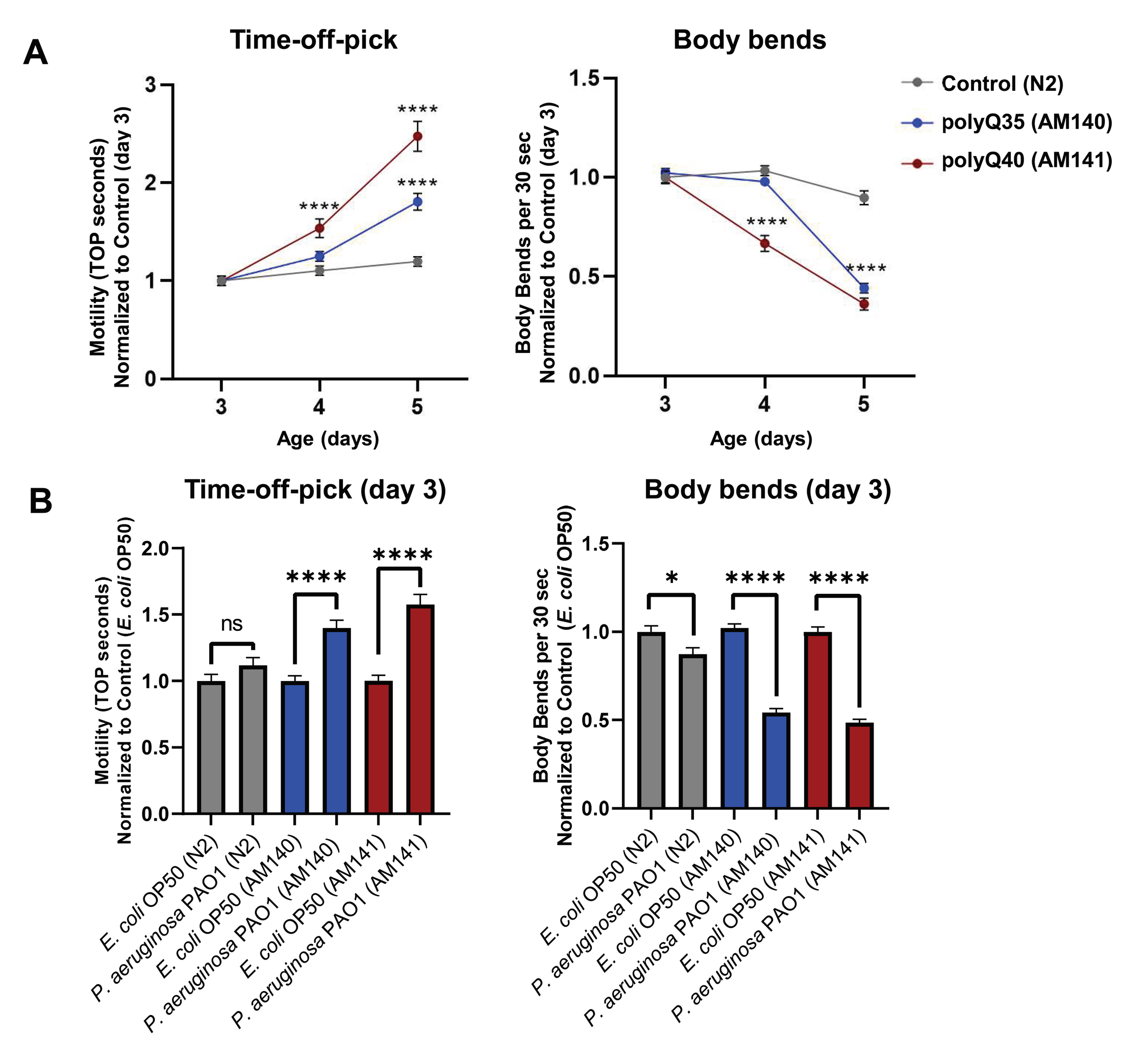

Supplement: S3 Fig — A) Age-dependent decline in motility assessed by increased TOP (left) and decreased number of body bends per 30 seconds (right) in muscle-specific polyQ35 (AM140), muscle-specific polyQ40 (AM141), and control (N2) worms. The data are represented as the average TOP seconds or average number of body bends per worm normalized to day 3. Each data point represents the average of two independent experiments with a total of 30 worms. B) The effect of bacteria on the motility at day 3 assessed by TOP (left) and body bends (right) in muscle-specific polyQ35, muscle-specific polyQ40, and N2 control worms. Data are represented as the average TOP or average number of body bends per worm normalized to animals fed E. coli OP50. Each bar is an average of two independent experiments with a total of 30 worms. Error bars represent SEM. Significance was calculated using one-way ANOVA followed by multiple comparison Dunnett’s post-hoc test (*p<0.05, ****p<0.0001). (TIF) [file ppat.1009510.s003.tif]

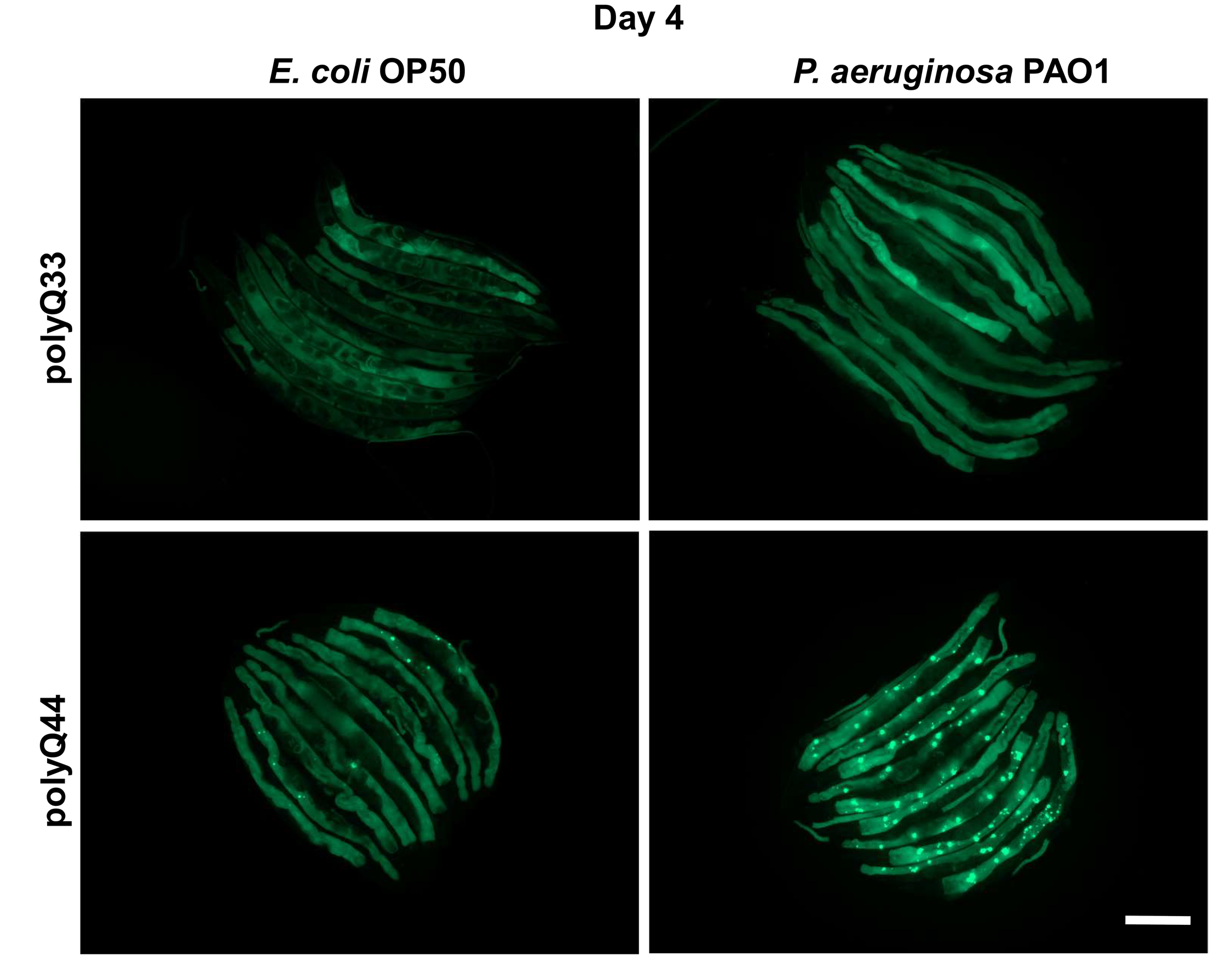

Supplement: S4 Fig — Fluorescent images represent worms expressing either polyQ33 or polyQ44 that were fed control E. coli OP50 or test strain P. aeruginosa PAO1 for a period of four days. Scale bar = 200 μm. (TIF) [file ppat.1009510.s004.tif]

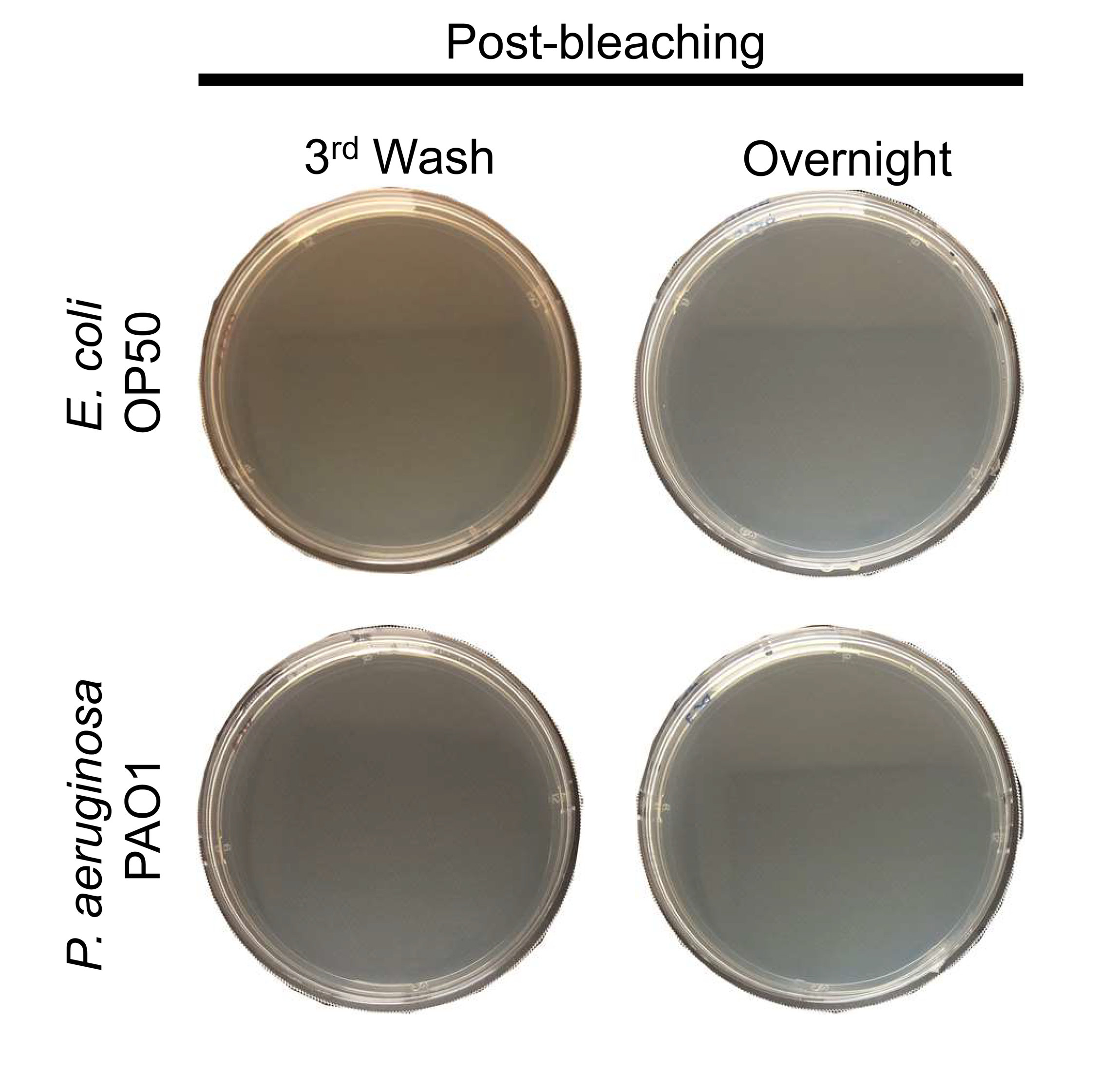

Supplement: S5 Fig — Images represent LB agar plates seeded with samples of M9 media from a 3rd wash of embryos post-bleaching and from overnight incubation of embryos that were allowed to hatch into L1 stage. Parental strains were colonized either with E. coli OP50 or P. aeruginosa PAO1. No colony-forming units were detected, indicating that all samples were void of bacteria. (TIF) [file ppat.1009510.s005.tif]

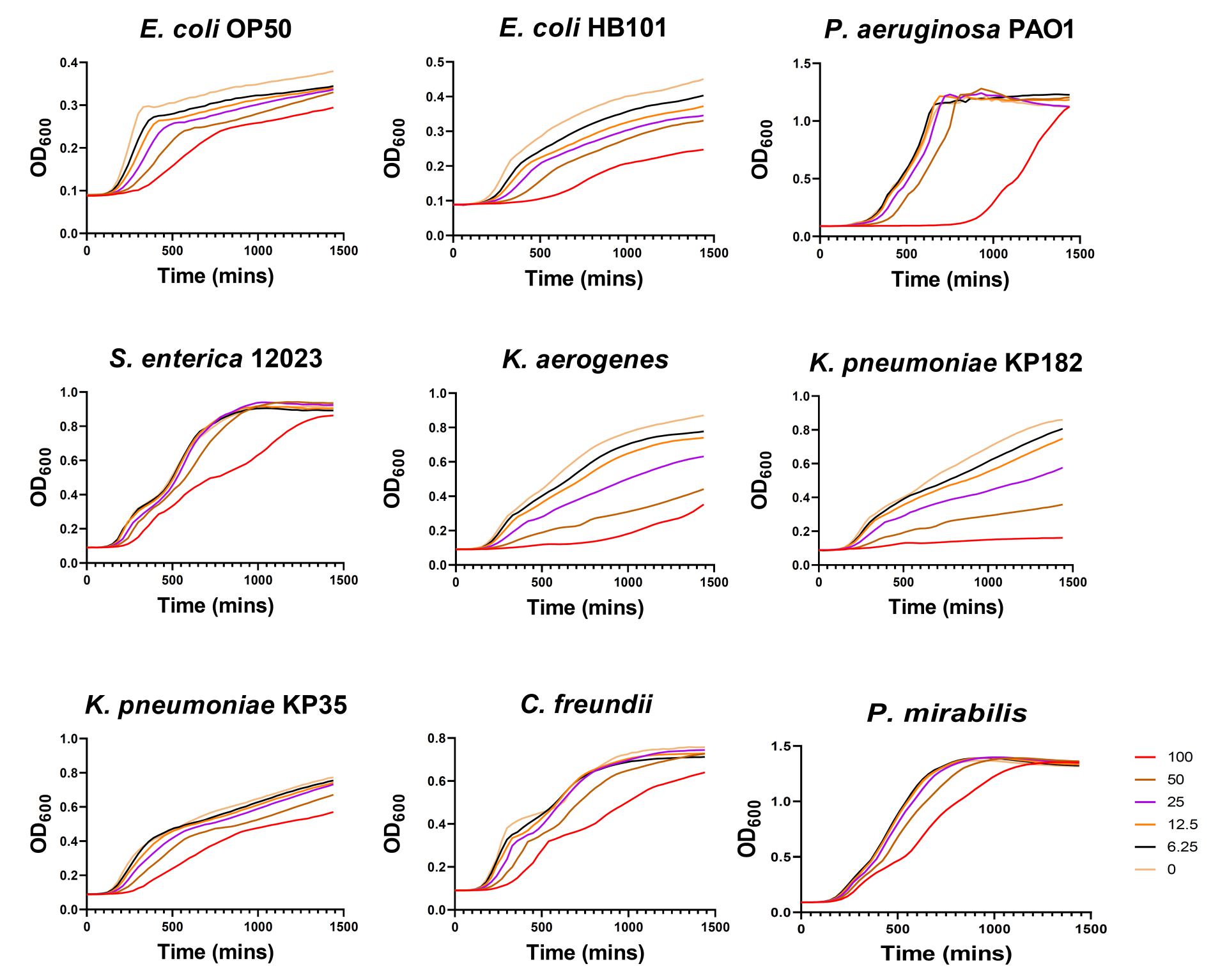

Supplement: S6 Fig — The growth of bacterial cultures was assessed in the presence of butyrate (0–100 mM) by measuring optical density at 600 nm (OD600). (TIF) [file ppat.1009510.s006.tif]

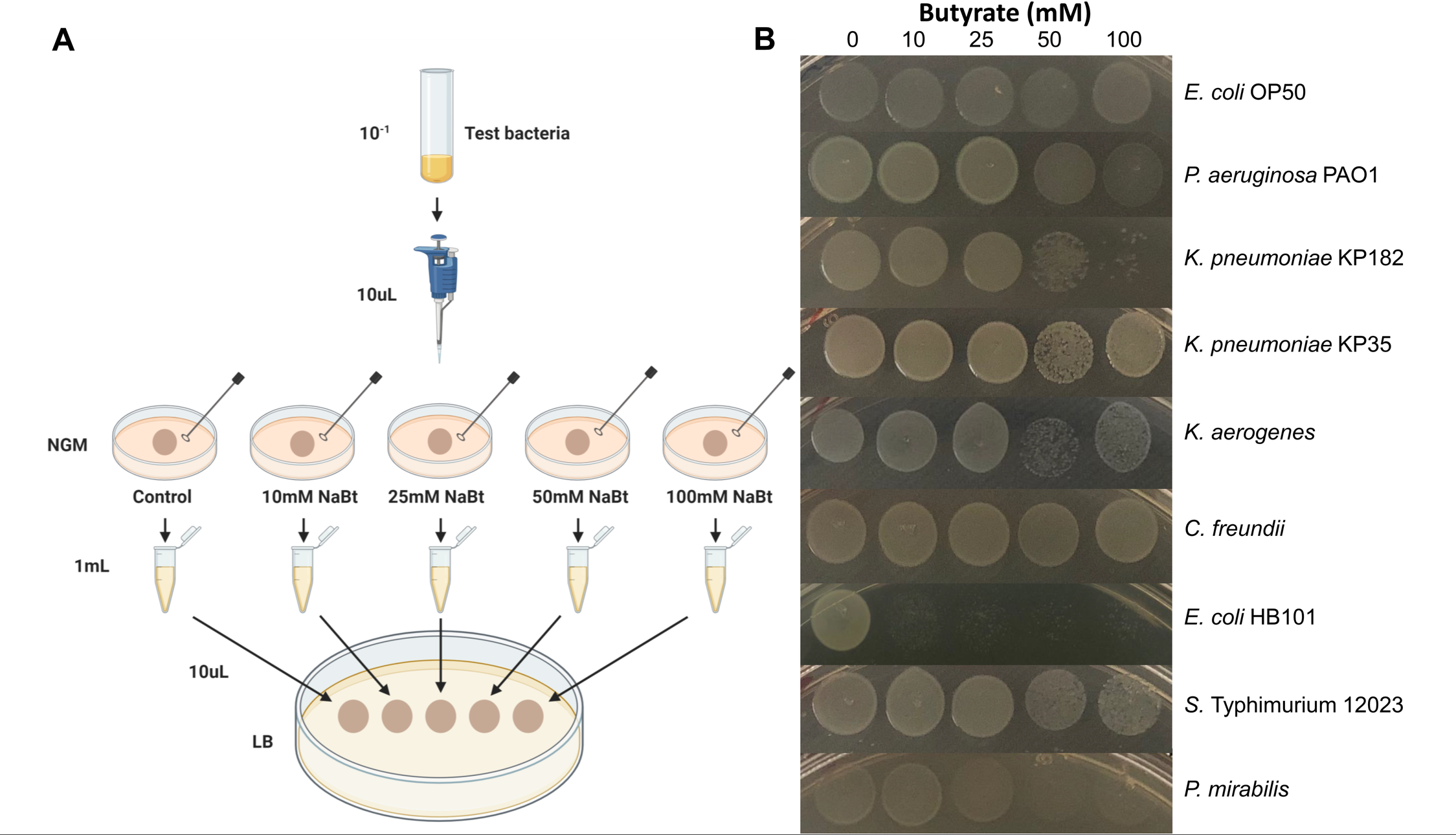

Supplement: S7 Fig — A) A cartoon depicting the procedure. B) Growth of bacteria collected from butyrate NGM plates and spotted on LB agar. (TIF) [file ppat.1009510.s007.tif]

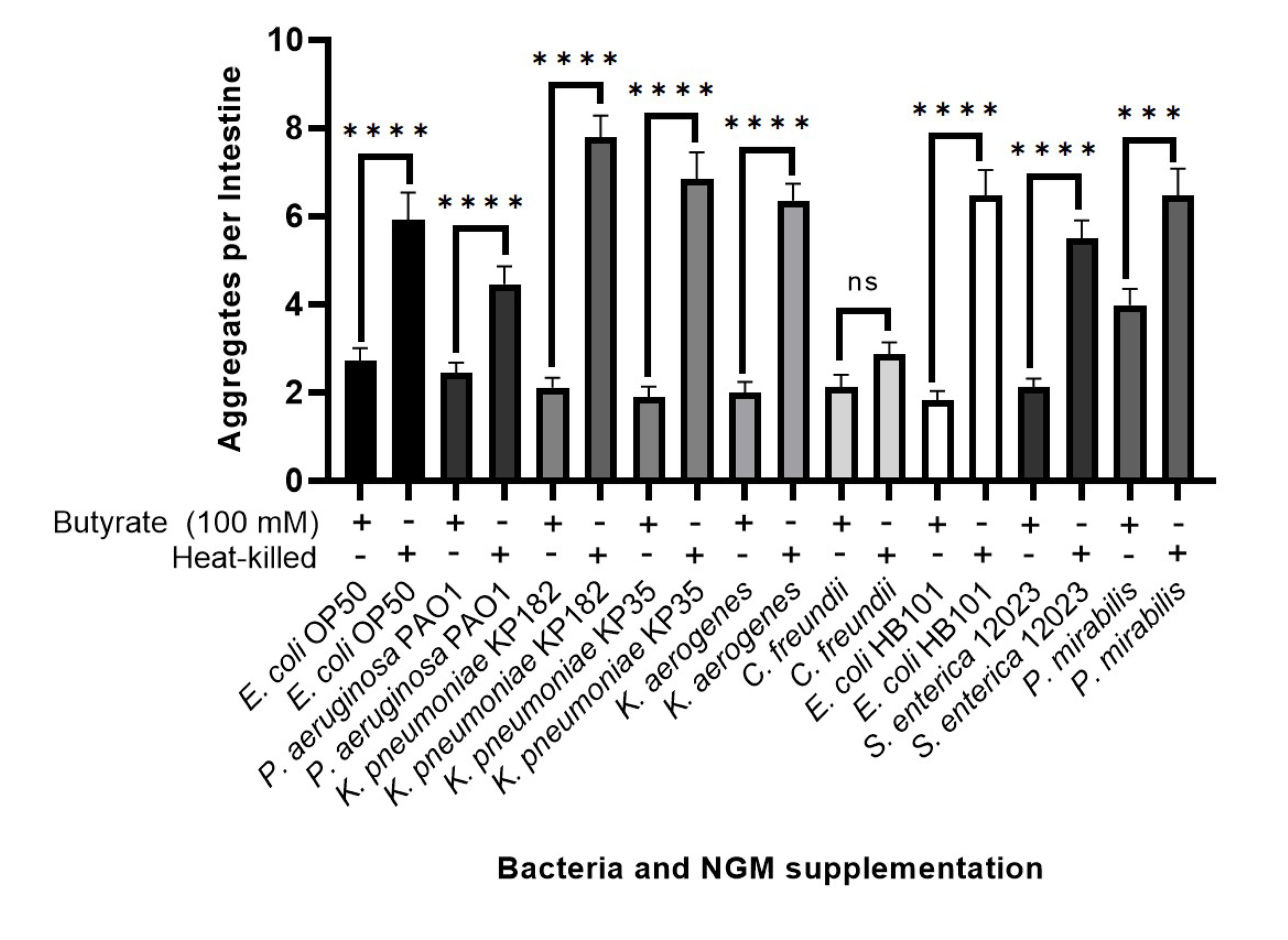

Supplement: S8 Fig — Data are represented as the average number of aggregates per worm. Each bar is an average of three independent experiments with a total of 100 animals. Error bars represent SEM. Statistical significance was calculated using Student’s t-test (ns: non-significant, ***p<0.0005, ****p<0.0001). (TIF) [file ppat.1009510.s008.tif]

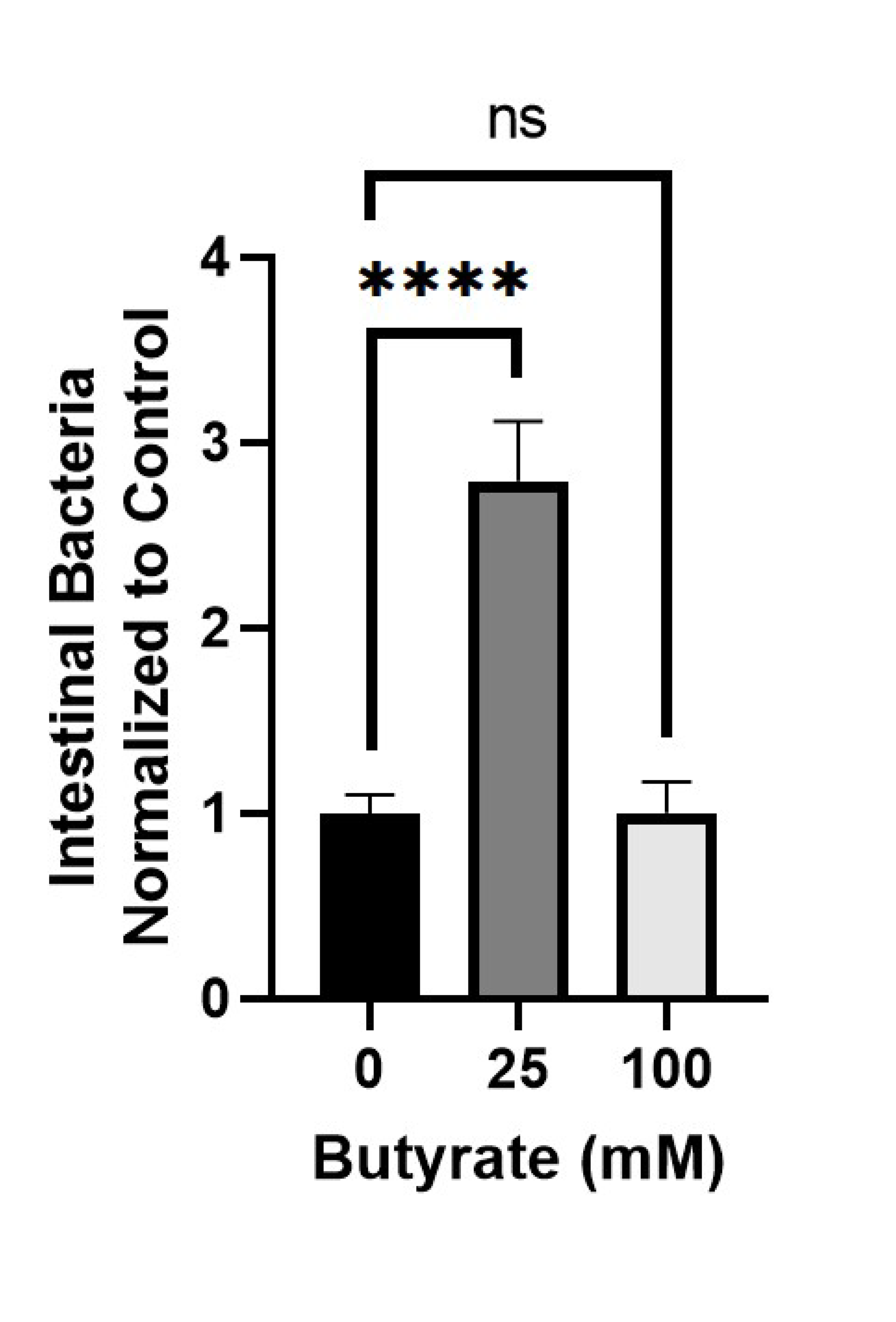

Supplement: S9 Fig — Bacterial load was enumerated by extracting intestinal E. coli OP50 on day four from animals expressing intestinal polyQ44 reporter (AM738). Data are represented as the average bacterial load per C. elegans intestine normalized to the control (0 mM butyrate). Each bar represents three independent experiments with a total of 30 animals. Error bars represent SEM. Significance was calculated using one-way ANOVA followed by multiple comparison Dunnett’s post-hoc test (ns: non-significant, ****p<0.0001). (TIF) [file ppat.1009510.s009.tif]

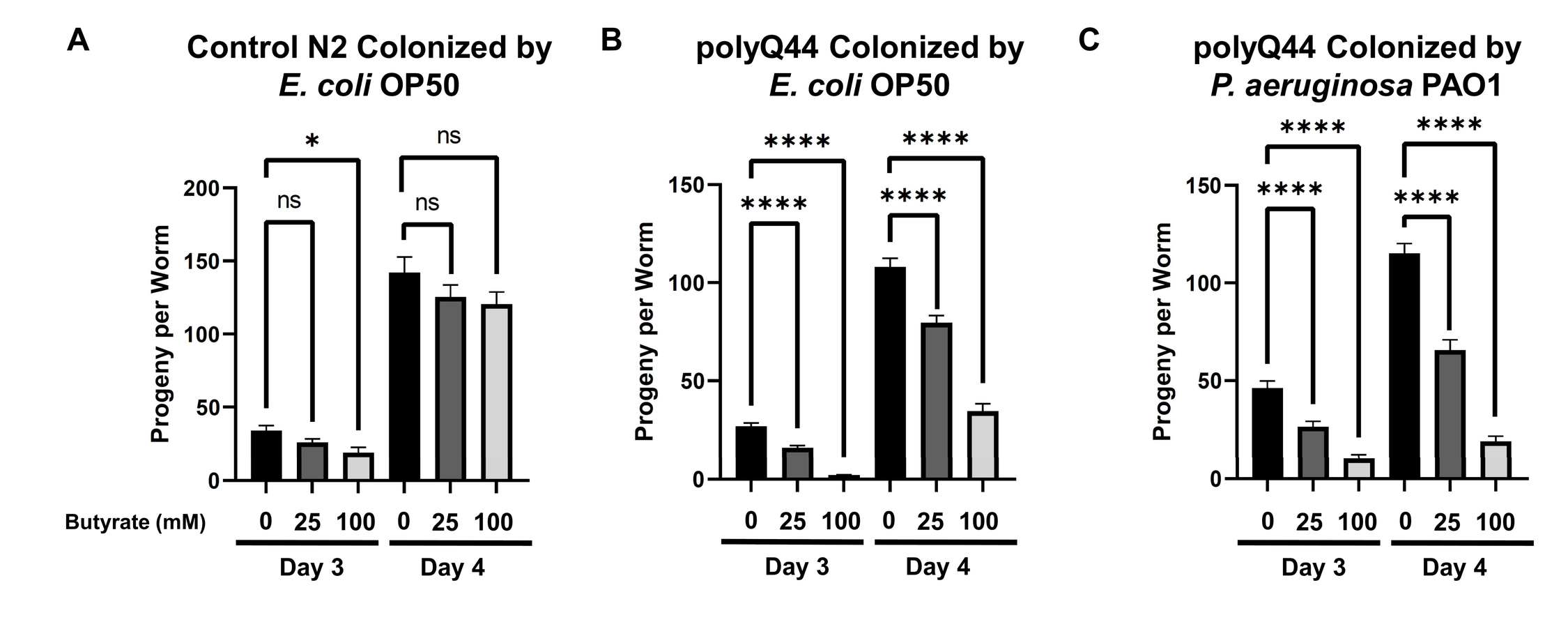

Supplement: S10 Fig — The effect of 0, 25, 100 mM butyrate on fecundity, days three and four, in: A) N2 worms colonized with E. coli OP50, B) polyQ44 colonized with E. coli OP50, C) polyQ44 colonized with P. aeruginosa PAO1. Each bar represents the average of three (B) and two (A, C) independent experiments with a total of 93 and 62 worms, respectively. Error bars represent SEM. Significance was calculated using one-way ANOVA followed by multiple comparison Dunnett’s post-hoc test (ns: non-significant, *p<0.05, ****p<0.0001). (TIF) [file ppat.1009510.s010.tif]

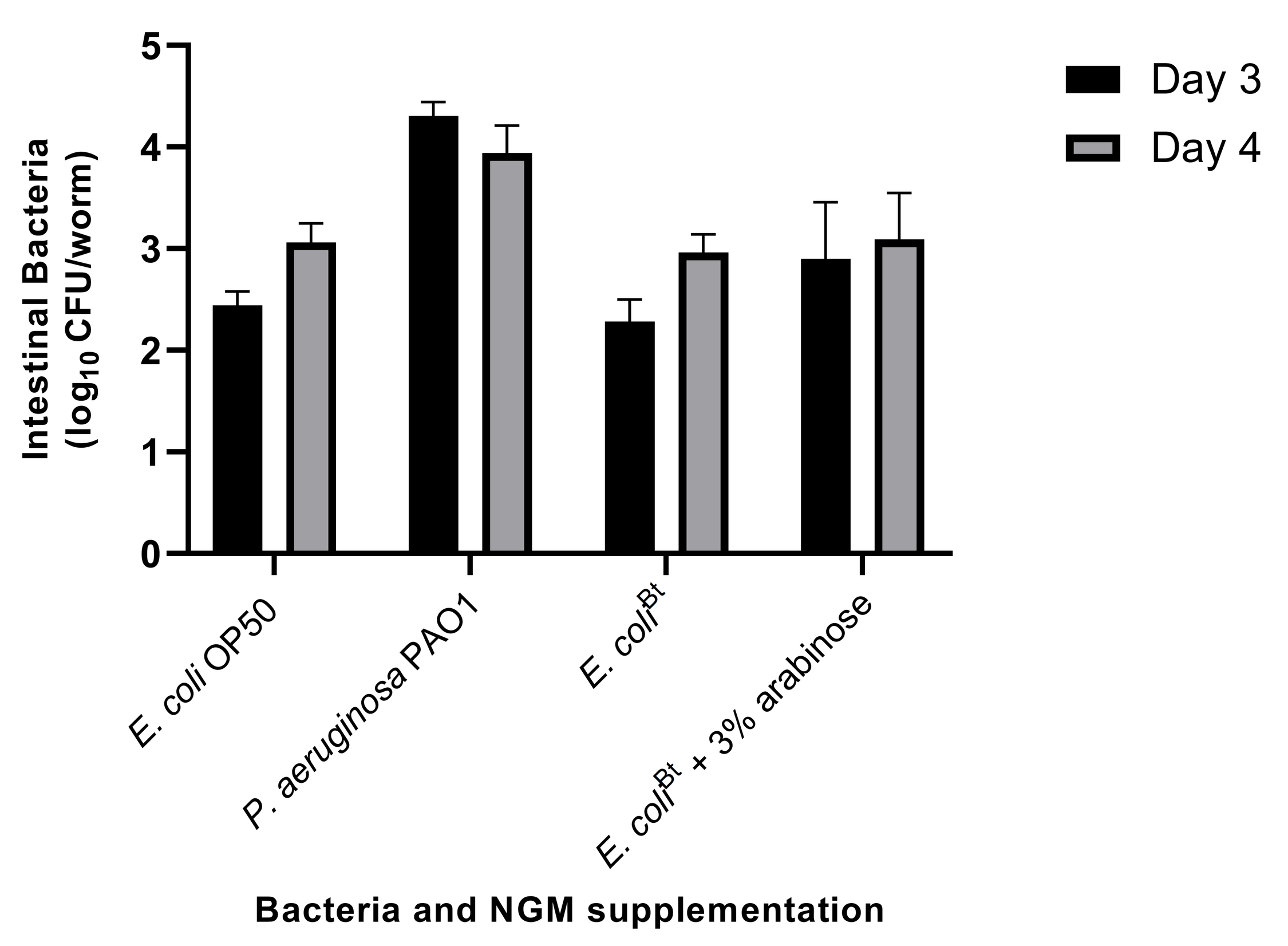

Supplement: S11 Fig — Bacterial load was enumerated by extracting intestinal bacteria (E. coli OP50, P. aeruginosa PAO1, E. coliBt +/- L-arabinose) on days three and four from animals expressing intestinal polyQ44 reporter (AM738). Each bar represents an average of three independent experiments with a total of 30 animals. Error bars represent SEM. (TIF) [file ppat.1009510.s011.tif]

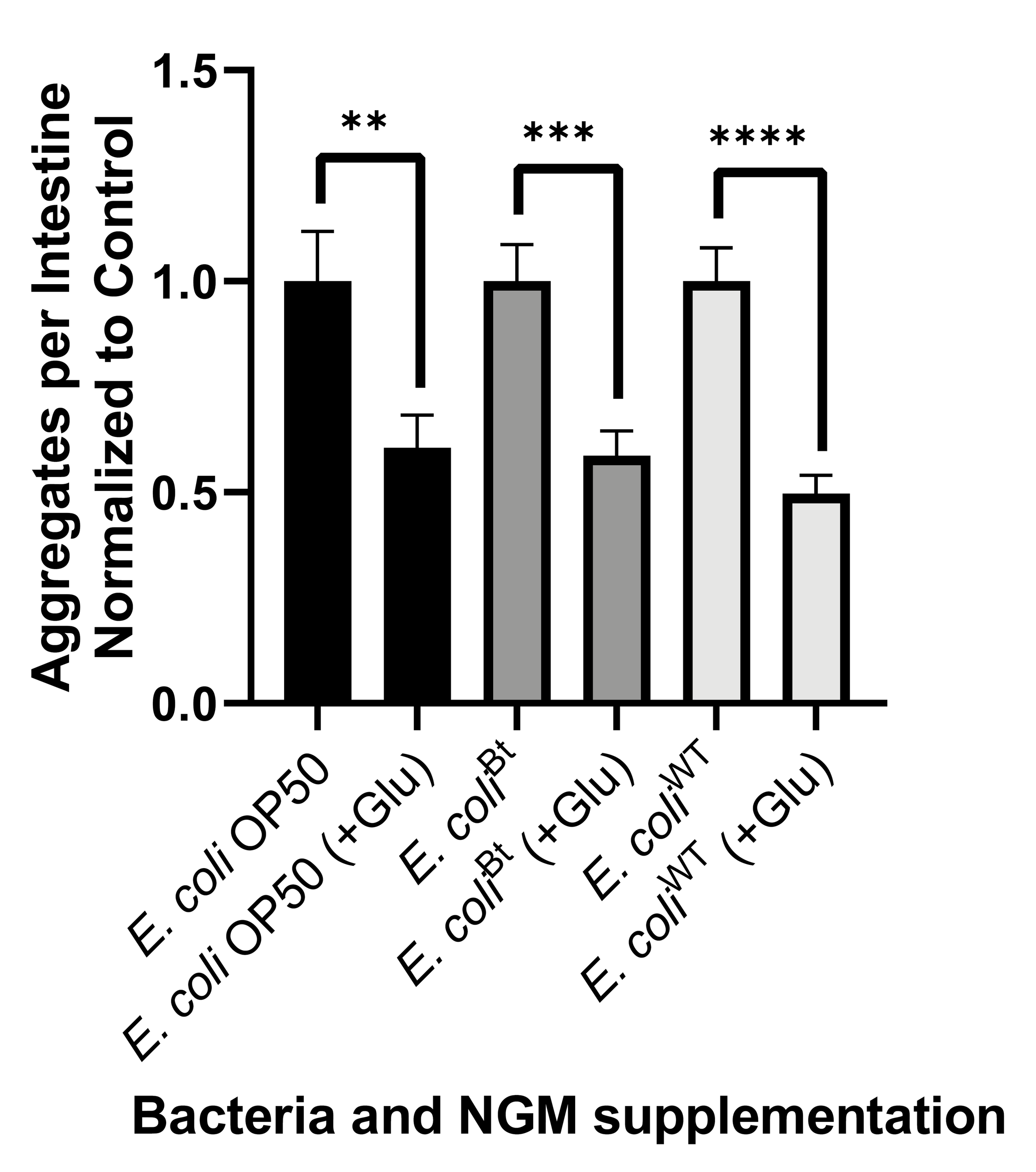

Supplement: S12 Fig — Data are represented as the average number of aggregates of intestine-specific polyQ44 (AM738) per worm normalized to the control (no glucose). Each bar is an average of two independent experiments with a total of 40 animals. Error bars represent SEM. Statistical significance between each pair was calculated using Student’s t-test (**p<0.01, ***p<0.001, ****p<0.0001). (TIF) [file ppat.1009510.s012.tif]

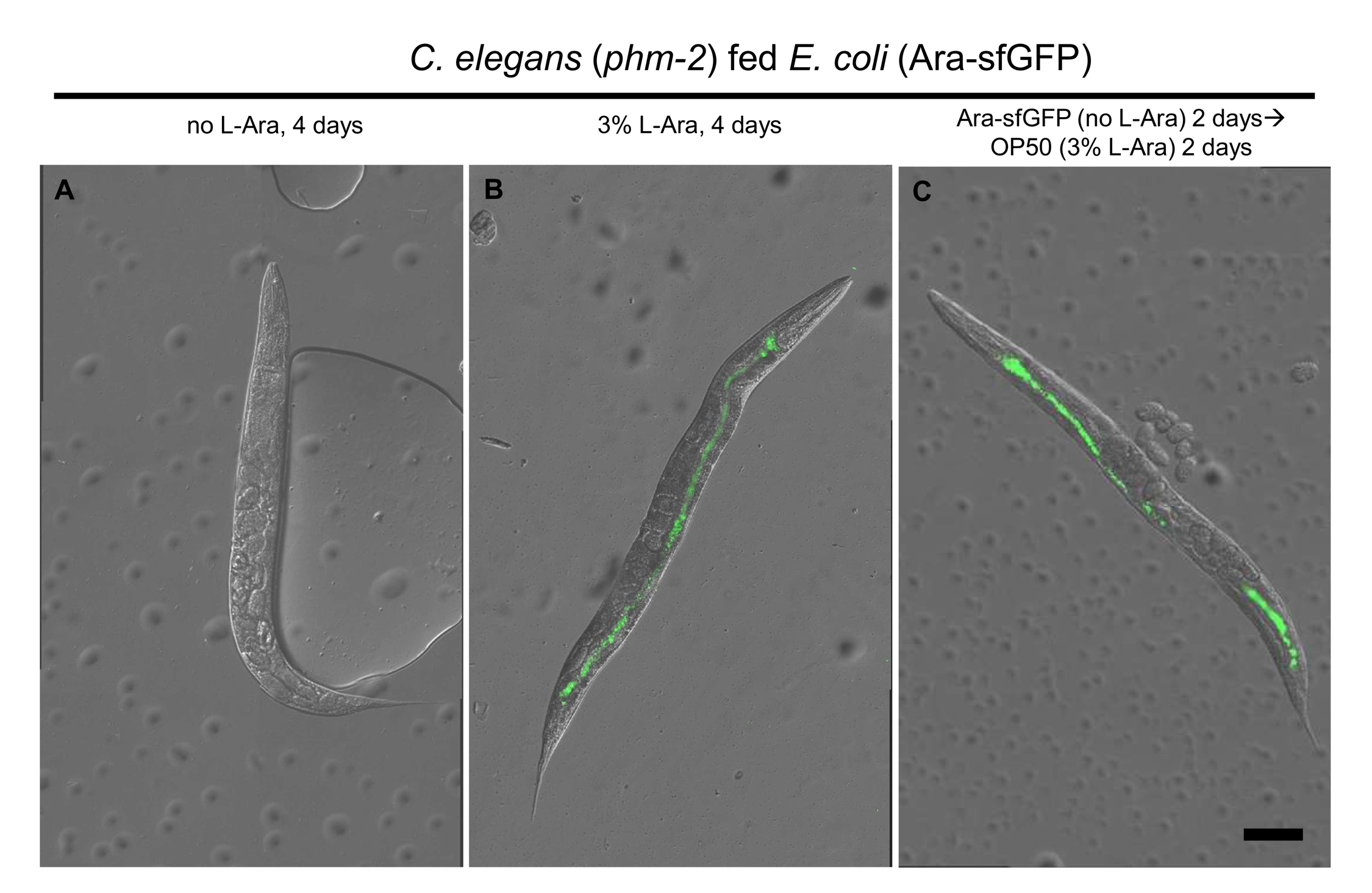

Supplement: S13 Fig — A) An overlay of Nomarski and GFP images of phm-2 worms cultured for four days on E. coli bacteria (DC228) carrying an L-arabinose-inducible reporter (Ara-sfGFP). B) Phm-2 worms cultured on E. coli bacteria in the presence of 3% L-arabinose. C) phm-2 worms cultured for two days on E. coli bacteria carrying L-arabinose inducible reporter in the absence of L-arabinose followed by a transfer onto 3% L-arabinose plates containing E. coli OP50. Scale bar = 200 μm. (TIF) [file ppat.1009510.s013.tif]
