# Supplementary material for: Colonization of the Caenorhabditis elegans gut with human enteric bacterial pathogens leads to proteostasis disruption that is rescued by butyrate
Source: PLoS Pathog. 2021 May 6;17(5):e1009510. doi: 10.1371/journal.ppat.1009510 (PMC8101752; doi:10.1371/journal.ppat.1009510)
Supplement: Attachment — Submitted filename: Review_response.docx [file ppat.1009510.s015.docx]

Reviewer's Responses to Questions

**Part I - Summary**
Please use this section to discuss strengths/weaknesses of study, novelty/significance, general execution and scholarship.

**Reviewer #1:** The human microbiome is suspected of influencing morbidity and mortality due to protein conformational diseases such as Alzheimer’s, Parkinson’s and Lou Gehrig’s diseases. However, the complexity of the human gut and inability to precisely control experimental conditions therein have hindered progress understanding the mechanisms underpinning these phenomena. Modeling microbiome connections to organismal protein homeostasis (proteostasis) has been successful in the nematode C. elegans, where ingestion of human pathogenic bacteria has been demonstrated to cause proteostatic collapse. In this report, the authors utilize the worm intestinal colonization approach paired with sensitive poly-glutamine-GFP proteostasis reporters to demonstrate that several important human pathogens disrupt proteostasis both immediately in the gut as well as in distal tissues including muscle and neuron. The short chain fatty acid butyrate has been shown to partially ameliorate such phenotypes in other studies, and indeed the same occurs here – exogenous or bacterially produced butyrate suppressed both polyQ aggregation and resultant phenotypes.

The work presented in this report is cleanly executed and the results largely clear and compelling. It teeters on the edge of incrementalism given the wealth of reports preceeding it on the topic, but breaks new ground with the butyrate experiments that clearly tie pathogen-induced proteostasis defects with previous findings. Although not fully explored, the observation that pathogen-induced proteostasis collapse extends into the F1 generation is striking. Additionally, the use of several pathogenic bacteria strongly reinforce the observations and lend credence to the model that pathogens disrupt protein quality control not just in local but also distal tissues, with profound ramifications for human biology. The only thing lacking is mechanistic insight into how butyrate is performing the observed rescues – more on this below. Overall this should be an important addition to the proteostasis, worm and neurodegeneration communities.

**Response:**

We thank reviewer #1 for valuable feedback. Based on the reviewer’s recommendation, we further explored the mechanism by which butyrate enhances host proteostasis and suppresses bacteria-mediated protein aggregation in the host. We found that the ability of butyrate to suppress bacteria-induced polyQ aggregation is dependent SKN-1, an ortholog of the mammalian Nrf2 transcription factor and DAF-16, an ortholog of the mammalian FOXO transcription factor involved in regulating the insulin-like signaling pathway. Heat shock factor 1 (HSF-1), a major transcriptional regulator of molecular chaperones, was not involved. Both SKN-1 and DAF-16 transcription factors are involved in responses to oxidative stress. Therefore, we believe that in part, bacteria may be disrupting host proteostasis through oxidative stress, which could be generated either by the bacteria directly or by the host. These results have been included in figures, results, and discussion. Finally, we provided additional data that help to decipher the role of bacteria on host proteostasis. We generated *E. coli* curli mutant strains and demonstrated that bacteria deficient in aggregates exhibit less effect on polyglutamine aggregation. These results reveal potential mechanisms by which bacteria may be affecting host proteostasis.

**Reviewer #2:** In this paper “Colonization of the Caenorhabditis elegans gut with human enteric bacterial pathogens leads to proteostasis disruption that is rescued by butyrate”, Czyz and colleagues evaluate the effects of exposing a panel of C. elegans strains expressing polyQ expansion proteins in the gut, muscle and neurons, to a panel of Gram-negative enteric bacterial pathogens. The goal of their studies is to understand the effects of the gut microbiome on protein aggregation and toxicity. The authors show that a diet of pathogenic bacterial strains predominantly exacerbates polyQ aggregation and impairs mobility of the animal, while exposing animals to bacteria that conditionally synthesize butyrate, suppresses this detrimental effect. The data are intriguing and suggestive that gut bacteria can influence protein aggregation not only in the gut, but also indirectly, the aggregation of protein in other tissues. However, the conclusions of the manuscript are still rather premature and descriptive, the non-autonomous effects described are not substantiated and could be due to less interesting reasons such as the health of the animals, and there are technical and conceptual issues which this reviewer thinks the authors should address before the paper is acceptable for publication.

**Response:**

We thank reviewer #2 for the feedback. We have performed additional control experiments and incorporated the corresponding results into our manuscript. The control experiments further eliminate the possibility that bacteria negatively influence *C. elegans* health so much so that illness itself is the cause for the non-autonomous effects observed across tissues. Additionally, we demonstrated that bacteria-derived aggregates influence host proteostasis, which suggests that specific bacterial components contribute to the observed increase in polyQ aggregation as opposed to general toxicity induced by these bacteria. Please see the responses below for more details.

**Reviewer #3:** Manuscript by Walker et al investigates how the intestinal colonization of C. elegans by enteric pathogens affect protein folding in the intestine and other systemic sites. Authors show that the colonization of the C. elegans intestine with enteric bacterial pathogens disrupts organismal proteostasis and enhances aggregation of polyQ and decrease motility. They use animals that constitutively express intestine-specific polyQs fused to yellow fluorescent protein (polyQ44::YFP) to quantify aggregation. They also use similar models to assess protein aggregation in muscles, neurons and gonads. Authors also show that butyrate can reverse polyQ aggregation and proteotoxicity and improve host proteostasis. Overall, this is a very interesting study and important for the understanding of how microbiota or pathogens can influence protein aggregation in the gut. There are some controls missing in the figures. I would highly recommend identifying whether the effect of enteric bacteria are inflammation driven or not. This could be achieved by comparing the commensal strains to pathogenic strains of enteric bacteria, or using sterile inflammation or avirulent mutants. I would also highly recommend testing the effect of butyrate on enteric bacteria as recommended below. Finally, recent studies suggested that curli expressing enteric bacteria can affect alpha synuclein aggregation. It would improve the impact of the manuscript if authors can address whether enteric curli mediates the polyQ aggregation in the gut.

**Response:**

We thank reviewer #3 for the feedback. We have added extra controls to the experiments that address the raised concerns. *C. elegans* lacks the inflammatory response, which is one of the reasons why we believe the bacteria-mediated effect on aggregation is more direct, as opposed to being driven by inflammation. However, we did address the reviewer’s concern by testing commensal bacteria from the human gut microbiome for their effect on protein aggregation. We assessed the effect of intestinal colonization by commensal bacteria on polyglutamine aggregation and found strains that either did not enhance aggregation or almost completely suppressed it. These results are very exciting as they further support our conclusions that enteropathogenic bacteria contribute to disruption of host proteostasis. Additionally, the two strains that suppressed aggregation in our model are from the *Prevotella* genus. The presence of these bacteria in Parkinson’s disease patients was shown to negatively correlate with the severity of the disease. Finally, we generated *E. coli* curli mutant strains and have demonstrated that a lack of bacterial protein aggregates lessens aggregation of polyglutamines, suggesting that bacterial aggregation-derived aggregates (or metastable proteins) contribute to the disruption of proteostasis in the host. We also added an extra section in our discussion that concentrates on the impact of bacterial products on host proteostasis. Please see the responses below for more details.

# **Part II – Major Issues: Key Experiments Required for Acceptance**

# Reviewer #1:

**Comment 1:** Previous work in worms and elsewhere has tied butyrate to inhibition of histone deacetylases and therefore transcriptional stress responses that may counteract the observed increase in protein aggregates. Given the tractability of the worm model and the ease of doing the experiments, it seems a missed opportunity for the authors to not try and connect the dots. I strongly suggest using DAF-16, SKN-1 and HSF-1 mutant worm lines to repeat the butyrate rescue experiments to determine whether one or more of these well-established proteostasis-supporting pathways are involved.

**Response:**

We appreciate that the reviewer provided such a helpful and constructive critique. In our original submission, we revealed a discovery that butyrate suppresses bacteria-induced polyglutamine aggregation in a concentration-dependent manner for some but not all bacterial strains (i.e., old Fig 3, new Fig 4). To determine whether the butyrate-dependent suppression of bacteria-mediated aggregation is dependent on the three transcription factors (HSF-1, DAF-16, SKN-1) that regulate major stress responses, we knocked-down each of them and assessed the beneficial effect of butyrate on polyglutamine aggregation. We found that the butyrate protective response is dependent on two transcription factors, SKN-1 and DAF-16, and updated the figures and text accordingly (please see below). Additionally, we confirmed the functional knockdown of each of these transcription factors. We thank the reviewer for suggesting these additional experiments that added a more mechanistic understanding to our study.

**We added the following text to our manuscript:**

***Note that all line numbers correspond to the MS Word document that includes tracked changes.***

**Lines 39-41:** Further experiments demonstrated that the beneficial effect of butyrate depended on the bacteria that colonized the gut and that this protective effect required SKN-1/Nrf2 and DAF-16/FOXO transcription factors.

**Lines 60-67:** Further experiments revealed that co-colonization with butyrogenic bacteria inhibited protein aggregation in *C. elegans* and the butyrate-mediated suppression of aggregation is dependent on SKN-1/Nrf2 and DAF-16/FOXO – two transcription factors involved in the regulation of oxidative stress responses. While the mechanism of bacteria-mediated induction of protein aggregation remains elusive, our results suggest that bacterial aggregates, in addition to the contribution of oxidative stress, are the contributing factor.

**Lines 364-386:** To gain a mechanistic understanding of how butyrate suppresses bacteria-mediated aggregation, we investigated the dependence of the observed response on three major evolutionary-conserved transcription factors, HSF-1, SKN-1/Nrf2, and DAF-16/FOXO. Each of these transcription factors was previously shown to provide protection against proteotoxicity (38-40). Because butyrate suppressed aggregation upon colonization with multiple strains of bacteria, including *E. coli* OP50 (**Fig 4**), we investigated the dependence of butyrate-mediated suppression of aggregation on the abovementioned transcription factors by knocking-down each candidate using RNAi. *C. elegans* carrying intestinal polyQ44 that were fed *E. coli* HT115 (DE3) expressing empty vector control (L4440) RNAi, exhibited a significant suppression of aggregation in the presence of butyrate (10-100 mM) (**Fig 7A**). While butyrate-mediated suppression of aggregation was not affected when HSF-1 was knocked-down, the beneficial effect of butyrate was abolished upon downregulation of SKN-1/Nrf2 and DAF-16/FOXO transcription factors. As expected, HSF-1 knockdown enhanced aggregation in the absence of butyrate when compared to control animals (**Fig 7A**) (41). To confirm that the knockdown is functional, we used fluorescent reporters to assess the expression of downstream target genes of HSF-1 (*hsp70p*::GFP also known as *C12C8.1p*::GFP) and SKN-1 (*gcs-1p*::GFP), and we used DAF-16 (DAF-16::GFP) fluorescent fusion (**Fig 7B**). The expression of all fluorescent reporters was attenuated upon knockdown of the respective target genes. Together, these results indicate that butyrate can suppress bacteria-induced polyQ aggregation across tissues and rescues aggregate-dependent toxicity in the host. Such protective effect of butyrate seems to be mediated by SKN-1 and DAF-16 transcription factors.

**Lines 500-511:** Our data indicate that the benefits of butyrate depend on SKN-1/Nrf2 and DAF-16/FOXO transcription factors, suggesting that butyrate may inhibit bacteria-induced aggregation by activating protective stress responses, in particular, oxidative stress responses. Indeed, bacteria are known to trigger oxidative stress, which can contribute to polyQ aggregation (23). It is possible that various bacteria introduce different levels of oxidative stress and the strongest contributors affect host proteostasis, consequently leading to misfolding and aggregation of metastable proteins present within the proteome. In fact, oxidative stress is one of the major contributors to PCDs (77). Recently, it was found that butyrate and β-hydroxybutyrate provide Nrf2- and FOXO-dependent protection against oxidative stress, respectively (78, 79). In addition, both SKN-1 and DAF-16 were shown to be involved in β-hydroxybutyrate-mediated lifespan extension (80). These results, along with our data, collectively suggest that butyrate activates SKN-1/Nrf2 and DAF-16/FOXO, which attenuate bacteria-induced oxidative stress. The effect of butyrate on polyQ aggregation also seems to depend on the type of pathogenic bacteria present, suggesting that butyrate may contribute to host proteostasis by acting on bacteria in addition to the host.

**Comment 2:** Fig. 8 (new Fig 10) is a little perplexing to this reviewer. The butyrate-producing E. coli appear to reduce aggregates in the control strain as much or more than they reduce it in the presence of P. aeruginosa. One interpretation is that the butyrate effect has nothing to do with the pathogen presence per se and is simply a physiological modulator of proteostasis in general. I suspect if these two experiments weren’t normalized, the aeruginosa effect would look much more dramatic, since the control strain should have few aggregates to start with.

**Response:**

Per reviewer's suggestion, we changed the y-axis of Fig 10A (old Fig 8A) to show the absolute number of aggregates per intestine. We want to further address the reviewer’s comment that: “One interpretation is that the butyrate effect has nothing to do with the pathogen presence per se and is simply a physiological modulator of proteostasis in general.” We agree that butyrate could affect general host proteostasis. From the literature, we know that butyrate targets host proteostasis (i.e., affects gene expression, provides cytoprotection, extends lifespan, suppresses aggregation in tissue culture, etc.) and from our new data, we also know that butyrate-mediated suppression of aggregation is dependent on DAF-16/FOXO and SKN-1/Nrf2, which are two transcription factors involved in oxidative stress responses. Certain bacteria may introduce various levels of oxidative stress (i.e., production of H_2_O_2_ by the bacteria, activation of innate immunity by the host, or both), resulting in different effects on host proteostasis. Such presence of reactive oxygen species would depend on the bacteria present. Therefore, the butyrate-mediated effect likely depends on bacteria. We have added additional experiments into our manuscript that demonstrate the involvement of bacterial-derived protein aggregates in the disruption of host proteostasis (Fig 1D-G, see below). These results suggest that bacteria-derived protein aggregates affect host proteostasis. Additionally, our results shown in Figure 4 (old Fig 3) demonstrate that at low levels, butyrate enhanced polyglutamine aggregation only if specific bacterial strains were present (i.e., *Pseudomonas*, *Salmonella*, or *Proteus* spp.) and at low concentrations, butyrate suppressed aggregation. As such, butyrate may enhance general proteostasis by activating protective stress responses (ex. DAF-16/FOXO and/or SKN-1/Nrf2), but based on our data, it is unlikely that such mechanism is the sole contributor. Together, these results support our conclusion that the cytoprotective effect of butyrate depends on the pathogen present.

# Reviewer #2:

**Comment 1:** The mechanisms by which pathogenic bacteria cause an increase in protein aggregation are not adequately addressed.

Are the animals dying earlier, sick or nutrient deprived, or

is the innate immune response activated and thus causing an increase in ROS-induced damage, etc.?

These questions need to, at the very least, be addressed in order to evaluate the significance or novelty of the tissue-nonautonomous influence (ln 137) that the authors highlight. If it is simply that intestinal cells are dysfunction, and therefore muscle cells are also nutrient deprived, it would be suggestive of one, perhaps less exciting mechanism. On the other hand, if the effects are due to hormonal signaling between the intestine and muscle, it would be suggestive of another, perhaps more intriguing mechanism.

**Response:**

We thank the reviewer for these suggestions. We have expanded our discussion section to provide a possible mechanism by which bacteria may contribute to polyQ aggregation in *C. elegans*. Also, to expand on the potential mechanisms involved in bacteria-mediated enhancement of protein aggregation, we explored the possibility that bacterial curli, aggregation-prone secreted amyloid proteins, contribute to the disruption of the host proteostasis and lead to polyQ aggregation. To test this, we generated *E. coli* strains that are deficient in curli (CsgA which encodes an amyloid structural subunit of curli fimbriae and CsgD, which is a global transcriptional regulator that positively regulates the curli operon), colonized *C. elegans* intestines with these strains, and assessed polyQ aggregation. As expected, both strains were deficient in amyloids as determined by Congo Red staining; however, only the global transcriptional regulator mutant (*ΔcsgD*) was deficient in bacteria-derived protein aggregates as assessed by ProteoStat, which is a fluorescent dye that has superior sensitivity in the detection of aggregates. This decrease of bacterial protein aggregates also corresponded to a decrease in intestinal polyQ aggregation upon colonization. These results support that bacteria-derived aggregates affect host proteostasis. We further explain that this could be the mechanism by which bacteria affect distant tissues.

**We added the following text to our manuscript:**

**Lines 159-170:** To begin understanding how bacteria could contribute to the disruption of host proteostasis, we used *E. coli* defective in the production of curli, which are aggregation-prone amyloids that are specific to *Enterobacteriaceae* (24)*.* We generated two mutant *E. coli* strains: one deficient in *csgA*, which encodes an amyloid structural subunit of curli fimbriae, and one deficient in *csgD*, a transcription factor that positively regulates the curli operon (**Fig 1D**). While staining with congo red confirmed a deficiency in curli production (**Fig 1E**), staining for bacterial protein aggregates using ProteoStat^®^, which provides superior sensitivity in aggregate detection (25), revealed that only *csgD* mutant cells have significantly fewer aggregates (**Fig 1F**). In agreement with these results, we found that colonization of *C. elegans* only with the Δ*csgD* *E. coli* strain led to a significant decrease in the number of polyQ aggregates per intestine (**Fig 1G**). These results suggest that bacteria-derived aggregates significantly contribute to the disruption of host proteostasis.

**Lines 581-595:** The introduction of metastable proteins is known to affect the stability and folding of other proteins within the proteome (70, 71). As such, it is likely that bacteria may be secreting factors that translocate from the intestine across other tissues and affect proteostasis. A cross-seeding between disease-associated proteins (i.e., Aβ) with bacterial amyloids, including those from *E. coli* and *P. aeruginosa*, was previously observed (72, 73). Also, an enhancement of alpha-synuclein aggregation mediated by bacterial curli was shown in mouse and *C. elegans* Parkinson’s disease models (74). While our results indicate that *E. coli* curli fimbriae were not responsible for the enhancement of polyQ aggregation, *E. coli* aggregates did play a contributing factor (**Fig 1G**). It is possible that alpha-synuclein and polyQ are affected differently by bacterial amyloids. In addition to regulating the *csg* operon, CsgD also functions as a global transcriptional regulator and its target gene(s), other than *csgA*, could affect host proteostasis (75). It is intriguing to speculate that the transmission of aggregation-prone proteins between tissues (67), may underlie how bacterial colonization of parental intestines affects protein aggregation in the progeny (**Fig 3I**).

The reviewer also brings an important point: “*If it is simply that intestinal cells are dysfunction, and therefore muscle cells are also nutrient-deprived, it would be suggestive of one, perhaps less exciting mechanism*.” While bacteria may be influencing the function of the *C. elegans* intestinal cells in a similar way that human gut microbiota affects intestinal cells, nutrient deprivation is unlikely the contributor to the observed enhancement of aggregation. Dietary restriction has been shown to enhance proteostasis, suppress aggregation and proteotoxicity, and extend lifespan. Therefore, if there is any nutrient deprivation, we would not expect to see suppression of aggregation and we would expect to see some developmental arrest; however, under our experimental conditions, none of the strains cause developmental arrest or any visible signs of animals being sick or dying early (although we have not done longevity experiments). In fact, we were able to plate *C. elegans* on test bacteria as L1 (first developmental stage) because none of the pathogenic bacteria in our experiments caused a developmental arrest. We have expanded on this topic more in the discussion section.

**We have the following text to our manuscript:**

**Lines 549-556:** Recent studies show that colonization of the *C. elegans* intestine with pathogenic bacteria affects bacterial avoidance behavior (63) and consequently increases lifespan (64). While it is possible that feeding behavior might have been affected in our experiments, it is unlikely that avoidance would contribute to an increase in polyQ aggregation and the associated motility defects. In fact, if the animals in our experiments experienced food avoidance behavior, we would expect to see enhanced proteostasis, as dietary restriction was previously shown to suppress polyQ aggregation (65).

Furthermore, our results in Fig 2B, Fig 2C, Fig 3B-C, and Fig 3E-F demonstrate that bacteria alone do not cause any detectable toxicity in control animals that do not express polyQ. Therefore, we know that bacteria contribute to polyQ aggregation, and the observed motility defect is dependent on the presence of polyQ aggregates.

**We added the following text to our manuscript:**

**Lines 487-488:** Importantly, our results demonstrate that bacteria alone do not cause major toxicity in control animals that do not express polyQ, which would hinder the interpretation of our data (**Fig 2B-C**, **Fig 3C**, **F**).

In the additional data that we provided in the revised version, we showed that SKN-1 and DAF-16 are required for butyrate-mediated suppression of aggregation. Because both of these transcription factors are involved in the regulation of oxidative stress responses, it suggests that in addition to bacterial-derived protein aggregates, bacteria may be inducing oxidative stress that contributes to polyQ aggregation, and butyrate mediates suppression of such oxidative stress. This conclusion is also supported by previous research showing that bacteria cause oxidative stress and contribute to aggregation in *C. elegans* (Mohri-Shiomi & Garsin, 2008), and sodium butyrate provides protection against oxidative stress (Sun et al. 2019).

**We added the following (highlighted) text to our manuscript:**

**Lines 523-528:** It is known that bacterial colonization of the *C. elegans* intestine increases the production of reactive oxygen species (ROS), which in turn enhances aggregation in that tissue (23). Oxidative stress is known to affect overall animal physiology, including motility (66). Since our results show the bacteria-mediated motility defects only in the presence of aggregation-prone polyQ44, but not in non-aggregating polyQ33 or non-polyQ controls (**Fig 2C**), it is possible that ROS generated by the worm in response to bacterial colonization enhanced polyQ aggregation, which further affected the function of other tissues involved in motility. Such a mechanism likely contributes to the observed bacteria-mediated disruption of host proteostasis which is supported by our results demonstrating that butyrate rescued polyQ aggregation in a DAF-16 and SKN-1-dependent manner (**Fig 7A**). However, ROS is likely not the sole contributor since our results also indicate that bacterial aggregates affect host proteostasis and consequently increase aggregation of host polyQ (**Fig 1E-G**).

**Comment 2:** The expression levels of polyQ under the different treatments should be measured to assess whether changes in protein expression explain the observed phenotypes.

**Response:**

Thank you for this suggestion. We performed western blot analysis of polyQ levels of both soluble protein and aggregates. Our results show that soluble protein levels do not significantly change between worms colonized with *E. coli* OP50, *P. aeruginosa* PAO1, in the presence of butyrate, or in the F1 progeny whose parents were colonized by either *E. coli* OP50 or *P. aeruginosa* PAO1. The insoluble fractions confirm our results obtained by quantifying fluorescent foci. These results are summarized in S1 Fig and S2 Fig (see below) and new text was added to the manuscript.

**We added the following text to our manuscript:**

**Lines 143-149:** To eliminate the possibility that bacteria change polyQ expression levels, using an antibody specific to YFP/GFP (**S1A Fig**), we confirmed that polyQ44:YFP soluble protein levels do not change between *E. coli* OP50 and *P. aeruginosa* PAO1 – two bacterial strains that elicited the lowest and highest intestinal aggregation, respectively (**S1B Fig**). Moreover, western blot analysis of the insoluble polyQ44::YFP fraction confirmed bacteria-mediated increase in aggregation (**S2A Fig**), as it parallels the aggregation profile of worms fed *E. coli* OP50 and *P. aeruginosa* PAO1 (**Fig 1B**).

**Lines 277-279:** Using western blotting, we ruled out the possibility that these results were due to changes in polyQ44::YFP expression level (**S1E Fig**) and we confirmed an increase of the insoluble polyQ44::YFP (**S2D Fig**).

**Lines 309-311:** We employed western blot analysis of polyQ44::YFP levels to eliminate the possibility that these results are due to changes in protein level (**S1C-D Fig**) and we confirmed these results by assessing insoluble fractions (**S2B-C Fig**).

**Comment 3:** The methodology associated with determining mobility is worrisome. The authors have devised a creative, and perhaps useful new method to assess mobility in animals harboring a roller phenotype (the TOP assay). However, no controls are included to address what is being measured using the TOP assay. Is the ability of animals to slide off an eyebrow hair a reflection of muscle contractility or some function of cuticle texture, hydrostatic pressure, eggs in the animal’s uterus, etc.? Controls using mutants known to be defective in some of these physiological features could provide more credence to the assay.

**Response:**

We performed additional experiments that support the application of our TOP assay. We used the TOP method to assess the motility of animals expressing muscle-specific polyQ35 and polyQ40. The age-dependent motility defect observed for these strains is known to be polyQ-specific (aggregation and motility defect is higher in polyQ40). As expected, using our new approach, we also detected age-related motility defect that is dependent on the polyQ presence and length (**S3 Fig**). These results agree with previously published motility defects observed with these strains (Morley et al. 2002). In addition to confirming that our TOP method can detect motility defects that are dependent on polyQ, we also performed body bend counts (**S3 Fig**), a well-established *C. elegans* motility readout frequently used with polyQ animals, and found that the effect of polyQs on body bends matched well the results that we obtained by measuring TOP.

**We added the following text to our manuscript:**

**Lines 184-196:** To ensure that the TOP method measures polyQ-specific defects and not an undesired effect related to the roller phenotype or bacterial pathogenicity, we used non-roller strains that express muscle-specific polyQ35 (*unc-54p*::Q35::YFP, AM140) and polyQ40 (*unc-54p*::Q40::YFP, AM141). Both of these strains are known to exhibit polyQ-length and age-dependent motility defects (29). In support of the functionality of our assay, we found that worms expressing these constructs also exhibited a significant polyQ-length and age-dependent TOP motility defect that correlated with the enumeration of body bends, which is a well-established assessment of motility in *C. elegans* (**S3A Fig**). Additionally, colonization of these polyQ-expressing strains with *P. aeruginosa* PAO1 inhibited motility in both assays yet had little to no effect in wild-type N2 animals. These findings indicate that polyQ toxicity, and not bacterial pathogenicity, is the factor that is measured by TOP (**S3B Fig**). Therefore, we further used the TOP method to determine whether colonization of the *C. elegans* intestine with bacteria that induced polyQ aggregation aggravates this phenotype.

**Comment 4:** The observation that the F1 progeny from infected mothers have higher aggregate loads is intriguing. Is protein synthesis altered in these animals? Do these animals express more polyQ proteins? Does bleach definitively remove all bacteria that the parents were in contact with?

**Response:**

We agree with the reviewer that it is crucial to demonstrate that polyQ levels in the F1 progeny do not change among animals whose parents were cultured on *E. coli* OP50 compared to those cultured on *P. aeruginosa* PAO1. We assessed polyQ::YFP protein levels by western blots and found no difference between progeny whose parents were cultured either on *E. coli* OP50 or *P. aeruginosa* PAO1. These results are included in **S1E Fig**. Additionally, we also confirmed that the insoluble fraction of polyQ44 increases in F1 progeny. These results are summarized in **S2D Fig**. F

**We added the following text to our manuscript:**

**Lines 277-279**: Using western blotting, we ruled out the possibility that these results are due to changes in polyQ44::YFP expression level (**S1E Fig**) and we confirm an increase of the insoluble polyQ44::YFP (**S2D Fig**).

Based on the reviewer’s suggestion, we eliminated the possibility that bacteria survive during synchronization and get in contact with the F1 progeny. We have accomplished this by 1) plating media from a third wash after the bleaching procedure and 2) plating media from overnight incubation of the embryos in the M9 medium. We did not detect any colony-forming units for *E. coli* OP50 nor *P. aeruginosa* PAO1. These results have been summarized in a new supplemental figure (**S5 Fig**).

**Comment 5:** It is known that decreasing fecundity has profound effects on polyQ aggregation and toxicity. Does butyrate affect fecundity? If so, depending on the mechanism, this may diminish the novelty of the observation.

**Response:**

Per reviewer’s suggestion, we performed experiments that look at the effect of butyrate on fecundity. Our results indicate that butyrate does affect fecundity and the effect on fecundity seems to be dependent on polyQ since the influence of butyrate on fecundity was more significant in animals expressing polyQ44 compared to wild-type N2 (S10 Fig). Previous reports showed that butyrate extended lifespan in wild-type N2 worms (Edwards et al. 2014); therefore, while fecundity may be a contributing factor, it is unlikely the explanation for the observed suppression of aggregation. Additionally, a decrease in fecundity alone does not explain the observed suppression of aggregation because at 25mM butyrate induced aggregation in worms colonized by *P. aeruginosa* PAO1(Fig 4, S2C Fig), despite a significant decrease in the average number of progeny (S10C Fig). Therefore, decreased fecundity could contribute to the suppression of aggregation, but is unlikely the sole contributor.

**We added the following text to our manuscript:**

**Lines 330-339:** A trade-off between organismal resources available to maintain either proteostasis or fecundity has been proposed (37). As such, we asked whether butyrate affects fecundity in our experiments, which may explain the suppression of aggregation. Indeed, we found that butyrate decreases the number of progeny per worm, but the effect seems to depend on the presence of polyQ (**S10A-B Fig**). Moreover, 25 mM butyrate significantly decreased the number of progeny in worms colonized by *P. aeruginosa* (**S10C Fig**). Yet, we found that under the same conditions, butyrate increased polyQ aggregation in animals colonized by *P. aeruginosa* (**Fig 4, S2C Fig**). These results indicate that a decrease in fecundity alone does not explain the observed suppression of polyQ aggregation.

**Comment 6:** In Figure 7 (new Fig 9), the authors should provide some way of rationalizing why polyQ animals fed E. coli expressing butyrate (E. coli Bt + arabinose) move more than wild-type animals exposed to the same conditions.

**Response:**

The reviewer brings an interesting point. At this moment, we do not have a reasonable explanation for this result. However, we did see similar results when worms expressing intestinal polyQ were colonized by *K. pneumoniae* or *P. aeruginosa* in the presence of 100 mM butyrate (Fig 5, left panel). Additionally, while the difference was not significant, we also detected a decrease in motility of control worms colonized by *E. coli*^Bt^ in the presence of arabinose (Fig. 8B, left panel)

**Comment 7:** Are developmental rates the same under all conditions?

**Response:**

We did not measure developmental rates; however, we did not observe any obvious changes in the development. For example, we performed the initial screen (Fig 1B) by placing worms on test bacteria at L1 developmental stage and assessed aggregation after four days. None of the worms arrested and all had similar size. In another study that is ongoing in our lab, we found a number of bacterial strains that significantly slow or arrest development; however, none of the bacteria studied in this project under the described conditions affected development.

8) This is applicable to multiple experiments: given the number of treatments, statistical analysis should be corrected for multiple comparisons (t-tests between two treatments alone can be misleading).

**Response:**

Thank you for pointing this out. We did use proper statistical analysis for each experiment, which was either a t-test or multiple comparison Analysis of Variance followed by Dunnett’s post-test when comparing multiple samples to the same control group. We might not have made it clear in the manuscript which test was done – we made sure to include statistical analysis statements in appropriate figure legends.

# **Reviewer #3:**

**Comment 1:** Can the commensal enteric bacteria influencing polyQ aggregation similar to pathogenic enteric strains? The authors can test this by comparing pathogenic versus commensal strains of couple of the bacteria tested here. It would tell us whether bacterial virulence mechanisms contribute to the polyQ aggregation. Alternatively, non-pathogenic or avirulent mutants of the bacterial strains can be used to as the same question.

**Response:**

We thank the reviewer for this great suggestion. In order to address this, we tested additional bacterial strains that are known to be part of normal bacterial flora. We colonized *C. elegans* intestines with each of the following bacteria: *Klebsiella* *oxytoca* HM-624, *Prevotella* *disiens* HM-1171, and *Prevotella* *corporis* HM-1294. While all of these strains are commensal, they could also become opportunistic pathogens. We found that *K. oxytoca* enhance aggregation in a manner similar to other *Klebsiella* spp. and both *Prevotella* spp. suppressed aggregation. In agreement with these results, we found previous reports that correlate a decrease in *Prevotella* abundance with enhanced pathogenicity of PCDs.

**We added the following text to our manuscript:**

**Lines 151-157:** We next asked whether gram-negative bacteria that are associated with commensal microflora would also enhance aggregation. We tested three strains known to be part of the commensal microbiome: *K. oxytoca* HM-624, *Prevotella disiens* HM-1171, and *Prevotella corporis* HM-1294. While *K. oxytoca* enhanced aggregation, as we have seen with the other *Klebsiella* spp., both *Prevotella* spp. suppressed aggregation relative to *E. coli* OP50 (**Fig 1C**). In fact, *P. corporis* almost completely suppressed aggregation.

**Lines 528-542:** However, both *P. disiens* HM-1171 and *P. corporis* HM-1294 suppressed aggregation relative to *E. coli* OP50 (**Fig 1C**). The mechanism by which these *Prevotella* spp. suppress aggregation does not depend on butyrate as both strains were deficient in its production (55, 56). Interestingly, *Prevotella* was the only bacterial genus whose abundance significantly decreased in patients suffering from constipation, a condition that is a hallmark in Parkinson’s disease and is associated with up to 80% of cases (57-59). Further evidence that these two species could be beneficial to host proteostasis comes from a recent study that demonstrated a negative correlation between the abundance of *Prevotella* in the gut of Parkinson’s disease patients and the severity of symptoms (60). A negative correlation between the abundance of *Prevotella* and pathogenesis of Alzheimer’s disease mouse model has also been observed (61). Collectively, these results suggest that the members of the *Prevotella* genus could play a more direct role in enhancing host proteostasis, and that the mechanisms by which bacteria affect host proteostasis in our *C. elegans* model may be conserved in higher organisms.

**Comment 2:** Can the authors elaborate more on the effect of butyrate? It would be a good addition to the manuscript at least to show if the same effect observed by bacteria can be recapitulated by a sterile inflammation by using low dose DSS? Earlier studies have shown that the supplementation of butyrate suppresses enteric bacterial expansion in the gut. This could be a similar mechanism here that the authors can show that the reversal of the inflammation may be leading to the reduction of the aggregation. If the authors can not use a chemical agent similar to DSS maybe they can plate the bacteria in Fig 8 and show that the numbers of enterics go down in the presence of butyrate.

**Response**

*C. elegans* lacks inflammatory response. This is one of the reasons why we think bacteria directly affect the host as oppose to indirectly through the induction of inflammation. We agree, it is possible that butyrate produced by commensal bacteria could suppress the growth of pathogenic bacteria, which contributes to the disruption of host proteostasis. In fact, we propose that such dysbiosis between enteric pathogens and commensal butyrogenic bacteria contributes to the pathogenicity of protein conformational diseases (Lines 107-108). We also added another piece of data which demonstrates that the suppression of aggregation by butyrate is not a result of decreased bacterial colonization (S9Fig). We saw an increase in the intestinal colonization by *E. coli* OP50 at 25 mM butyrate and no effect on colonization at 100 mM.

**We added the following text to our manuscript:**

**Lines 324-328:** Next, we asked whether butyrate could affect colonization of the intestine. We found that at 25 mM, butyrate significantly enhanced intestinal colonization by *E. coli* OP50, but exhibited no effect on colonization at 100 mM (**S9 Fig**). These results indicate that it is unlikely that the butyrate’s effect on bacterial colonization affects aggregation.

**Lines 601-604:** Our results demonstrate that butyrate, a common metabolite produced by commensal microbiota, can suppress aggregation and the associated toxicity when supplied exogenously (**Fig 4, Fig 6**) or produced by intestinal bacteria (**Fig 8, Fig 9**). We also showed that butyrate did not simply inhibit intestinal colonization (**S9 Fig**).

**Comment 3:** Although the effect of microbiota on host proteostasis could be attributed to systemic inflammation, recent studies have shown that the curli amyloid secreted by E. coli and other enteric bacteria can directly seed alpha synuclein deposition in mouse model of PD (Sampson et al 2020 eLife). Can authors comment on whether curli expressed in the C. elegans gut can effect the proteostasis in their model system? I would highly recommend adding an E. coli curli + and – or a S. Typhimurium curli+ and – strains to their assays to improve this knowledge gap and increase the impact of the paper.

**Response:**

We thank the reviewer for this great suggestion. We generated two *E. coli* mutant strains: *csgA*, an amyloid structural subunit of curli fimbriae and *csgD*, a transcription factor that positively regulates the curli operon. We confirmed the lack of amyloid fibrils using Congo Red staining for both of these strains; however, we saw a significant reduction (by ~50%) of polyQ aggregation only in worms that were colonized by the *csgD* mutant strain. We used a more sensitive assay (ProteoStat®) to detect aggregates and found that only the *csgD* mutant strain produced less protein aggregates. It is important to note that these aggregates were not related to curli amyloid. Together these additional results that are summarized in Figure 1D-F suggest that bacteria-derived aggregates contribute to the disruption of host proteostasis and ultimately affect polyQ aggregation.

**We added the following text to our manuscript (same as the response to Reviewer’s #2 Comment #1):**

**Lines 159-170:** To begin understanding how bacteria could contribute to the disruption of host proteostasis, we used *E. coli* defective in the production of curli, which are aggregation-prone amyloids that are specific to *Enterobacteriaceae* (24)*.* We generated two mutant *E. coli* strains: one deficient in *csgA*, which encodes an amyloid structural subunit of curli fimbriae, and one deficient in *csgD*, a transcription factor that positively regulates the curli operon (**Fig 1D**). While staining with congo red confirmed a deficiency in curli production (**Fig 1E**), staining for bacterial protein aggregates using ProteoStat^®^, which provides superior sensitivity in aggregate detection (25), revealed that only *csgD* mutant cells have significantly fewer aggregates (**Fig 1F**). In agreement with these results, we found that colonization of *C. elegans* only with the Δ*csgD* *E. coli* strain led to a significant decrease in the number of polyQ aggregates per intestine (**Fig 1G**). These results suggest that bacteria-derived aggregates significantly contribute to the disruption of host proteostasis.

**Lines 581-595:** The introduction of metastable proteins is known to affect the stability and folding of other proteins within the proteome (70, 71). As such, it is likely that bacteria may be secreting factors that translocate from the intestine across other tissues and affect proteostasis. A cross-seeding between disease-associated proteins (i.e., Aβ) with bacterial amyloids, including those from *E. coli* and *P. aeruginosa*, was previously observed (72, 73). Also, an enhancement of alpha-synuclein aggregation mediated by bacterial curli was shown in mouse and *C. elegans* Parkinson’s disease models (74). While our results indicate that *E. coli* curli fimbriae were not responsible for the enhancement of polyQ aggregation, *E. coli* aggregates did play a contributing factor (**Fig 1G**). It is possible that alpha-synuclein and polyQ are affected differently by bacterial amyloids. In addition to regulating the *csg* operon, CsgD also functions as a global transcriptional regulator and its target gene(s), other than *csgA*, could affect host proteostasis (75). It is intriguing to speculate that the transmission of aggregation-prone proteins between tissues (67), may underlie how bacterial colonization of parental intestines affects protein aggregation in the progeny (**Fig 3I**).

**Part III – Minor Issues: Editorial and Data Presentation Modifications**
Please use this section for editorial suggestions as well as relatively minor modifications of existing data that would enhance clarity.

# **Minor issues**

**Reviewer #1:** 
**Comment 1:** Suggest that the titles of figure panels be removed and only stated in the legends. The figure layout resembles a slide presentation more than a figure set at this point.

**Response:**

We modified labels in figure titles according to the reviewer’s suggestion. We did leave a few graph titles to make results more clear when different polyQ lengths were compared.

**Comment 2:** The polyQ reporters differ depending on the tissue being interrogated. While clear to aficionados, the reasoning behind these choices is not presented in the text. For example, Q33 is a control in Fig. 1, and Q35 an experiment in Fig. 2. A little more background would help.

**Response:**

Thank you for bringing this up. We clarified the choice of these reporters in the text.

**We added the following text to our manuscript:**

**Lines 215-220:** We employed nematodes expressing muscle-specific polyQ35 (*unc-54p*::Q35::YFP) as a proxy to detect changes in proteostasis in that tissue. Different tissues have different thresholds for polyQ aggregation; therefore, while polyQ33 did not aggregate in the intestine, polyQ35 aggregates in the muscle. This reporter exhibits age-dependent and muscle-specific aggregation and the associated motility defect (29).

# **Reviewer #2**

**Comment 1:** To show that arabinose causes the expression of butyrate the authors have used a phm-2 mutant strain. It is my understanding that the remaining experiments were not conducted in this background. The authors should explain the rationale for this difference, and also whether this difference impacts their remains experiments.

**Response:**

Our initial intention was to perform all of the experiments with polyQ44 in the *phm-2* mutant background. That way, we may be able to increase intestinal colonization. This was a good idea, but we failed to get a successful cross after numerous attempts. While *phm-2* background might have enhanced the observed phenotypes, we still obtained good intestinal colonization and phenotypes in the wild-type background. We ended up using the *phm-2* mutant strain to enhance intestinal colonization with bacteria that express arabinose-inducible GFP (Ara-sfGFP, DC228) and can facilitate the induction of fluorescence by arabinose supplemented in NGM plates. The *phm-2* background in this experiment did not affect any of the result outcomes. It simply enhanced colonization which allowed us to obtain a strong GFP signal. This experiment was only used to demonstrate that arabinose is accessible to bacteria colonizing the *C. elegans* gut.

# **Reviewer #3**

**Comment 1:** Fig 1A. No bacteria control is missing. It would be informative to see the kinetics of aggregation without E. coli. It is hard to say if there is any accelerated aggregation.

**Response:**

*C. elegans* go through four larval stages (L1-L4) after which they become gravid adults. These nematodes also have two developmental checkpoints (L1 and L2 – Dauer stage) and will arrest in the presence of stress or absence of food. Therefore, we cannot use “no food” controls in these experiments.

**Comment 2:** Fig1B. Can the authors comment on the species that didn’t show any aggregation? This information is not included in the results section. It would be informative to know if this strain is a commensal strain where as the others are pathogenic.

**Response:**

Thank you for this comment. We were able to generate exciting new data on strains that did not enhance aggregation. In fact, we found strains that suppressed aggregation with respect to our *E. coli* OP50 control. Please see the results and discussion section (below) which is a similar response provided to Reviewer #3 Comment #1.

**We added the following text to our manuscript:**

**Lines 141-157:** The only strain that did not significantly induce aggregation relative to *E. coli* OP50 was *E. coli* HB101, a non-pathogenic strain derived from *E. coli* K-12. To eliminate the possibility that bacteria change polyQ expression levels, using an antibody specific to YFP/GFP (**S1A Fig**), we confirmed that polyQ44:YFP soluble protein levels do not change between *E. coli* OP50 and *P. aeruginosa* PAO1 – two bacterial strains that elicited the lowest and highest intestinal aggregation, respectively (**S1B Fig**). Moreover, western blot analysis of the insoluble polyQ44::YFP fraction confirmed bacteria-mediated increase in aggregation (**S2A Fig**), as it parallels the aggregation profile of worms fed *E. coli* OP50 and *P. aeruginosa* PAO1 (**Fig 1B**). These results are intriguing as they demonstrate that common human enteric pathogens significantly affect polyQ aggregation, and therefore host proteostasis. We next asked whether gram-negative bacteria that are associated with commensal microflora would also enhance aggregation. We tested three strains known to be part of the commensal microbiome: *K. oxytoca* HM-624, *Prevotella disiens* HM-1171, and *Prevotella corporis* HM-1294. While *K. oxytoca* enhanced aggregation, as we have seen with the other *Klebsiella* spp., both *Prevotella* spp. suppressed aggregation relative to *E. coli* OP50 (**Fig 1C**). In fact, *P. corporis* almost completely suppressed aggregation.

**Lines 525-542:** On the other side of the aggregation spectrum, the non-pathogenic *E. coli* HB101 did not significantly enhance aggregation in our *C. elegans* polyQ model. The *E. coli* HB101 strain is also a common laboratory food for *C. elegans*; hence, we did not expect any enhancement with these bacteria. However, both *P. disiens* HM-1171 and *P. corporis* HM-1294 suppressed aggregation relative to *E. coli* OP50 (**Fig 1C**). The mechanism by which these *Prevotella* spp. suppress aggregation does not depend on butyrate as both strains were deficient in its production (55, 56). Interestingly, *Prevotella* was the only bacterial genus whose abundance significantly decreased in patients suffering from constipation, a condition that is a hallmark in Parkinson’s disease and is associated with up to 80% of cases (57-59). Further evidence that these two species could be beneficial to host proteostasis comes from a recent study that demonstrated a negative correlation between the abundance of *Prevotella* in the gut of Parkinson’s disease patients and the severity of symptoms (60). A negative correlation between the abundance of *Prevotella* and pathogenesis of Alzheimer’s disease mouse model has also been observed (61). Collectively, these results suggest that the members of the *Prevotella* genus could play a more direct role in enhancing host proteostasis, and that the mechanisms by which bacteria affect host proteostasis in our *C. elegans* model may be conserved in higher organisms.

**Comment 3:** To draw the conclusion that the polyQ aggregation is necessary for the pathogen-induced effect, the authors should repeat the aggregation experiment both in PolyQ33 and polyQ44 background. This wwould support the results obtained in Fig 1E .

**Response:**

This is a good suggestion. However, we decided not to use polyQ33 in the aggregation experiment, because this polyglutamine tract is under the aggregation threshold, and as a result, this strain does not exhibit aggregation even with aging (Mohri-Shiomi & Garsin, JBC 2008). We performed additional experiments and added a supplemental figure that shows a lack of aggregates in worms expressing polyQ33 colonized by *P. aeruginosa* PAO1, which is the most potent inducer (SFig 4).

**We added the following text to our manuscript:**

**Lines 204-208:** While it is known that worms expressing polyQ33 do not exhibit age-dependent aggregation, we wanted to ensure that aggregation does not occur when these animals are colonized by bacteria that induce polyQ44 aggregation. We did not detect polyQ33 aggregates in animals colonized with the strongest inducer, *P. aeruginosa* PAO1 (**S4 Fig**).

**Comment 4:** Fig 3 (new Fig 5). Have the authors considered plating the bacteria straight from the nematodes to see if butyrate effects the in vivo numbers? It is possible that the effect of the butyrate maybe stronger in vivo due to additional factors compared to in vitro treatment.

**Response:**

Thank you for this suggestion. We did not check the effect of butyrate on the colonization of all strains, but we added additional data only on the control *E. coli* OP50, as butyrate also decreased aggregation in a dose-dependent manner in worms colonized with this strain. Therefore, we can use these data to more clearly illustrate the presence or absence of a relationship between colonization and aggregation. We extracted and enumerated bacteria (*E. coli* OP50) from animals that were cultured on 0, 25, and 100 mM butyrate. We did not detect suppression of bacterial colonization. As described in one of the responses above, we saw an increase in intestinal colonization by *E. coli* OP50 at 25 mM butyrate concentration and no effect on colonization at 100 mM (S9 Fig).

**We added the following text to our manuscript:**

**Lines 314-317:** Next, we asked whether butyrate could affect colonization of the intestine. We found that at 25 mM, butyrate significantly enhanced intestinal colonization by *E. coli* OP50, but exhibited no effect on colonization at 100 mM (**S9 Fig**). These results indicate that it is unlikely that the butyrate’s effect on bacterial colonization affects aggregation.

**Lines 601-604:** Our results demonstrate that butyrate, a common metabolite produced by commensal microbiota, can suppress aggregation and the associated toxicity when supplied exogenously (**Fig 4, Fig 6**) or produced by intestinal bacteria (**Fig 8, Fig 9**). We also showed that butyrate did not simply inhibit intestinal colonization (**S9 Fig**).

**Comment 5:** Fig 3 (new Fig 5). Does butyrate treatment effect aggregation without the bacteria? No bacteria controls are missing.
**Comment 6:** Fig 4 (new Fig 6). Does butyrate treatment effect motility without the bacteria? No bacteria controls are missing.

**Response:**

Thank you for these suggestions; however, *C. elegans* model has some intrinsic limitations. As explained in one of the responses above, we cannot use “no food” controls in these experiments. *C. elegans* arrest development in the absence of food.

**Additional changes made by the authors:**

- We added new co-authors to our manuscript.
- Room temperature was changed from 25 to 23 ^o^C.
- Figure legends were added to all new respective figures.
- We realized that the results under “Gonad” section describing polyQ aggregation in the F1 generation might not be too clear to a non-*C. elegans* person; therefore, we modified this section to provide a more clear explanation of what was done.
- Any additional text that was added is marked with “Track Changes” in the revised manuscript.
